# Comparison of intensity‐modulated radiotherapy and forward‐planning dynamic arc therapy techniques for prostate cancer

**DOI:** 10.1120/jacmp.v9i4.2783

**Published:** 2008-10-24

**Authors:** Mohamed Metwaly, Awaad Mousa Awaad, El‐Sayed Mahmoud El‐Sayed, Abdel Sattar Mohamed Sallam

**Affiliations:** ^1^ Radiation Physics Department, Faculty of Science Ain Shams University Cairo Egypt; ^2^ Radiotherapy Department, Oncology and Hematology Hospital, Maadi Armed Forces Medical Compound, Faculty of Science Ain Shams University Cairo Egypt; ^3^ Physics Department, Faculty of Science Ain Shams University Cairo Egypt

**Keywords:** intensity modulation, dynamic arc, prostate cancer, radiation dosimetry

## Abstract

We compare an inverse‐planning intensity‐modulated radiotherapy (IMRT) technique with three previously published forward‐planning dynamic arc therapy techniques and a newly implemented technique for treatment of prostate only. The three previously published dynamic arc techniques are dynamic arc therapy (DAT), two‐axis dynamic arc therapy (2A‐DAT), and modified dynamic arc therapy (M‐DAT). The newly implemented technique is the bilateral wedged dynamic arc (BW‐DAT). In all dynamic arcs, the multileaf collimator is moving during rotation to fit the prostate, except that, in 2A‐DAT, it is fitting two separate symmetrical rhombi including the prostate. The rectum is shielded during rotation only in the cases of M‐DAT and BW‐DAT.

The results obtained indicate that the BW‐DAT, M‐DAT, and DAT techniques provide the intended dose coverage of the prescribed dose to the planning target volume (PTV)—that is, 95% of the PTV is covered by 100% of the dose. The maximum dose to a 3‐cm margin of healthy tissue that surrounds the PTV is lower by 2.5% in the case of IMRT than in both BW‐DAT and M‐DAT, but it is lower by 5.0% than that in both DAT and 2A‐DAT. The maximum dose to the rest of the healthy tissue in the case of BW‐DAT is 33.2Gy±2.2Gy. This dose covers percentage healthy body volumes of 8%±3.2% with IMRT, 4%±1.5% with DAT, and 6%±1.2% with both 2A‐DAT and M‐DAT. Also, this dose is much lower than the accepted maximum dose (52 Gy) to the femoral heads and necks according to Report 62 from the International Commission on Radiation Units and Measurements. Accordingly, it would be possible to neglect delineation of the femoral heads and necks as organs at risk in cases of BW‐DAT.

Doses to 15%, 25%, 35%, and 50% ($D_{15\%}$, $D_{25\%}$, $D_{35\%}$, and $D_{50\%}$) of the rectum volume in the case of BW‐DAT were 43.5Gy±8.6Gy, 24.2Gy±8.7Gy, 13.2Gy±4.2Gy, and 5.7Gy±2.1Gy respectively. The $D_{15\%}$ of rectum in the case of IMRT was lower than that in BW‐DAT, M‐DAT, 2A‐DAT, and DAT by 7.3%, 10.3%, 33.0%, and 17.6% of the prescribed dose (78 Gy in 39 fractions) respectively. The $D_{25\%}$, $D_{35\%}$, and $D_{50\%}$ of the rectum volume in the cases of IMRT and DAT were comparable (with a maximum variation of 4.5%); they were similarly comparable in the cases of M‐DAT and BW‐DAT (with maximum variation of 1.5%). These same doses in BW‐DAT were lower than those in IMRT by 8.7%, 10.6%, and 6.2% respectively, but they were quite lower than those in 2A‐DAT, because the average variation was 41.6% (with a maximum of 44.0%).

The $D_{15\%}$, $D_{25\%}$, $D_{35\%}$, and $D_{50\%}$ of the bladder volume in the case of BW‐DAT were 33.2Gy±10.9Gy, 17.4Gy±7.9Gy, 6.5Gy±4.3Gy, and 4.2Gy±3.5Gy respectively. The $D_{15\%}$ and $D_{25\%}$ of the bladder in the cases of IMRT, M‐DAT, and BW‐DAT were comparable (with a maximum variation of 2.2% and 3.6% respectively), and the mean values of each dose were lower in DAT by 14.3% and 11.7% respectively. However, the values of $D_{35\%}$ and $D_{50\%}$ in the four techniques were comparable, with maximum variations of 5.1% and 2.7% respectively. The $D_{15\%}$, $D_{25\%}$, $D_{35\%}$, and $D_{50\%}$ of the bladder in the case of DAT were lower than those in 2A‐DAT by 20.1%, 26.9%, 16.0%, and 2.7% respectively.

Ion chamber measurements showed good agreement between the calculated and measured isocentric doses (maximum deviation: 3.2%). Accuracy of the dose distribution calculation for BW‐DAT was evaluated by film dosimetry using a gamma index, allowing 3% dose variation and 3 mm distance to agreement as the individual acceptance criteria. We found that fewer than 6.5% of the pixels in the dose distributions of the scanned and calculated area of 10×10 cm failed the acceptance criteria.

We conclude that, in addition to simplicity of the dose calculation, the BW‐DAT technique provides the intended concave dose distribution for treatment of the prostate only. Compared with IMRT, it produces better dose protection to the most of the rectum volume and to the healthy tissue outside the treatment volume. Also, as compared with the other forward planning dynamic arc techniques, it gives the most favorable isodose distributions to the prostate and rectum.

PACS number: 87.53.Tf

## I. INTRODUCTION

In radiation therapy, a maximum dose must be delivered to the tumor while the dose to the surrounding normal tissues and organs at risk is minimized. Three‐dimensional conformal radiation therapy (3D‐CRT) is a technique designed to deliver prescribed radiation doses to localized tumors with high precision, while using a multileaf collimator (MLC) to effectively exclude the surrounding normal tissues. In the forward planning process, as used in 3D‐CRT, the MLC is set to shape the radiation fields using various angles (gantry angles) to conform to the tumor or planning target volume (PTV), and the dose weighting is then adjusted in a trial‐and‐error fashion to refine the plan. The only feasible way to change the radiation intensity in 3D‐CRT is to use static or dynamic wedges (which achieve intensity modulation through a single line in the beam aperture plane) and compensating filters (metallic plates of non‐uniform thickness, which achieve intensity modulation in the all points of the field aperture plane). However, preparation and treatment delivery sessions with the use of compensating filters take a long time and bear a risk of human error.

Intensity‐modulated radiotherapy (IMRT) with dynamic MLC is the logical extension of 3D‐CRT, in which each of the radiation fields is divided into thousands of finite‐size pencil beams of varying intensities by means of an inverse planning process. In this process, the user sets dose constraints that permit a maximum dose to the PTV and restrict the dose to the adjacent risk structures. The planning computer, through numerous iterations, comes up with the best possible intensities for the pencil beams and a dynamic MLC movement that together realize the dose constraints. As a result, dose intensity modulation occurs in all points of the plane of the beam aperture. Compared with 3D‐CRT, IMRT delivers equivalent or higher doses to the prostate with greater sparing of rectum, bladder, and femoral heads in prostate cancer radiotherapy.^(1)^

In parallel to IMRT, dynamic arc therapy was used as an effective alternative technique for prostate cancer radiotherapy.^(2)^ ^,^ ^(3)^ Dynamic arc therapy is a radiation therapy delivery technique also based on forward‐planning dose calculation, which combines gantry rotation with MLC motion to conform to the beam eye‐views of the PTV at various gantry angles.

Two techniques of bilateral dynamic arcs had been recently introduced and compared with IMRT for prostate cancer radiotherapy.^(2)^ ^,^ ^(3)^ In the first technique, 100 bilateral dynamic arcs (DAT) are used. The second technique involves two‐axis dynamic arc therapy (2A‐DAT) with half rotation (180 degrees) around two isocenters each in two separate symmetrical rhombi. The idea was to produce anterior and posterior concave distributions to protect the bladder and rectum by considering two symmetrical rhombi including the PTV as the target volume for the dynamic MLC instead of the PTV itself. The 2A‐DAT technique was found to provide sparing of normal structures equivalent to that of IMRT, although PTV dose uniformity was inferior. It was concluded that DAT and 2A‐DAT are possible alternatives to IMRT for prostate cancer radiotherapy.^(2)^ ^,^ ^(3)^

On the other hand, a simplified intensity‐modulated arc therapy (SIMAT) technique appeared as a modality of DAT for treatment of the prostate plus seminal vesicles with differential doses in three treatment phases.^(4)^ In SIMAT, a third‐phase treatment was added to handle treatment of the prostate only, in which the rectum was shielded by the MLC during rotation. Modified dynamic arc therapy (M‐DAT) was introduced as an extension of SIMAT.^(5)^ This technique uses full arcs that fit to the prostate plus seminal vesicles and that shield the rectum, combined with two lateral posterior oblique wedged conformal fields of low weighting that fit to the prostate only. It was concluded that, as compared with the three‐phase SIMAT technique, a single M‐DAT treatment phase produces less bladder protection in the region of lower doses with consistently greater sparing of the rectum in regions of higher and lower doses.^(5)^

In the present work, we are proposing a bilateral wedged dynamic arc therapy (BW‐DAT) technique to achieve a concave dose distribution to the prostate only. This technique uses two bilateral pairs of half rotation (180 degrees) around wedged arcs that fit the prostate only and shield the rectum. The main difference between BW‐DAT and M‐DAT is that the dose conformity to the prostate in BW‐DAT is achieved by the function of the hard wedge in the bilateral arcs instead of the two lateral posterior oblique wedged conformal fields in M‐DAT.

The BW‐DAT technique is implemented mainly to acquire as steep as possible a dose gradient outside the prostate, particularly in the rectum region. This approach avoids undesirable hot spots or localized high‐dose regions outside the treatment volume that would occur in cases of IMRT.

Also in the present work, we compared IMRT with M‐DAT, DAT, 2A‐DAT, and BW‐DAT to determine if any of these techniques provide advantages in sparing of rectum, bladder, femoral heads, and healthy tissue, with similar or better coverage of the prescribed dose to the prostate only. The accuracy of the dose calculation algorithm of our planning system for BW‐DAT was determined by comparison with measurements.

## II. MATERIALS AND METHODS

### A. Planning systems and radiotherapy machine

We used a BrainScan stereotactic 3D planning system (ver. 5.21: BrainLAB, Feldkirchen, Germany) for inverse‐planning dose calculation of IMRT plans with a micro‐multileaf collimator (m3: BrainLAB) of 52 leaves (26 pairs) of 3 mm thickness at the field central axis at the isocenter plane, then 4.5 mm farther out, and 5.5 mm at the field boundaries. An Eclipse 3D planning system (ver. 7.3.10: Varian Medical Systems, Palo Alto, CA) was used for forward‐planning dose calculation of dynamic arc plans with an 80‐leaf (40 pairs) multileaf collimator of 10 mm thickness at the isocenter. (Our Eclipse system is not licensed for IMRT and our BrainScan system is not licensed for dynamic arc therapy.) A Varian 23 EX linear accelerator was used for treatment delivery sessions using the BW‐DAT technique.

### B. Computed tomography and magnetic resonance imaging scans and volume definition

For 10 patients of interest, we performed computed tomography (CT) and magnetic resonance imaging (MRI) scans in the supine position with a slice spacing of 3 mm. The scan borders were taken through the region from the lower end of the sacroiliac joint down to the penile urethra plus 1 cm inferiorly and superiorly. The CT and MRI images were transferred electronically to the Eclipse and BrainScan systems, in which image fusion was performed.

In the two planning systems, the prostate (PO), rectum (RC), and bladder (BL) were contoured on MRI images, reviewed in CT images, and verified by the same radiation oncologist. For simplicity, and because all of the dynamic arc techniques are symmetrical with respect to laterals, only one femoral head and neck (FH) was delineated in CT images. For IMRT, both FHs were delineated. The margins for the PO were taken 10 mm in the superior, inferior, and anterior directions, 7 mm in the left–right direction, and 5 mm in the posterior direction.^(^ ^6^ ^–^ ^14^ ^)^ The resulting PTV for the PO was designated PPO.

The RC was taken through the region from the sigmoid colon superiorly to the anal canal inferiorly, including the rectal wall and cavity. The BL was delineated as the whole bladder. The average volumes of the PPO, RC, and BL for the 10 patients, as calculated by the two planning systems, were 120.2 cm^3^ (ranging from 80.2 cm^3^ to 168.5 cm^3)^, 170.6 cm^3^ (ranging from 80.2 cm^3^ to 205.4 cm^3)^, and 220.6 cm^3^ (ranging from 177.2 cm^3^ to 340.5 cm^3)^ respectively.

Instead of the RC, a visual rectum volume (VRV) was delineated to be shielded in the dynamic arc techniques so as to minimize the effect of the MLC penumbra on the PPO coverage, with adequate RC shield. This rectum volume was delineated to be contracted away from the target, in the anterior–posterior direction, by 10 – 12 mm.

In addition to the PPO, RC, BL, and FH, two volumes were delineated for estimation of the dose to healthy tissue. The first was a margin of healthy tissue around the PPO, which was used for estimation and comparisons of the volumes covered by high doses (>70% of the prescribed dose) in this region. A margin of 3 cm (3cmM) was found to be sufficient to include the isodose lines of the high doses. The sum of the PPO and 3cmM was considered the treatment volume (TV). The second volume (Body–TV) was the entire scanned volume (Body) minus the TV. This volume was used for estimations and comparisons of the doses outside the TV. Finally, each delineated volume was calculated by the two planning systems and compared to ensure equivalence.

### C. IMRT technique

An arrangement of 7 coplanar beams at gantry angles 0, 51, 102, 153, 207, 258, and 309 degrees with 6‐MV photon energy was used for IMRT.^(^ ^15^ ^–^ ^20^ ^)^ The dose constraint to the PPO was 100% of the PPO covered by 95% of the prescribed dose (78 Gy). The dose constraints of organs at risk followed the dose guidelines designed by the Radiation Therapy Oncology Group (RTOG) for patients being treated for localized prostate cancer under RTOG protocol 0126.^(21)^ The rectal criteria in the RTOG guidelines require that no more than 15%, 25%, 35%, and 50% of the rectum volume should receive more than 75 Gy, 70 Gy, 65 Gy, and 60 Gy respectively. The bladder criteria in the guidelines require that no more than 15%, 25%, 35%, and 50% of the bladder volume should receive more than 80 Gy, 75 Gy, 70 Gy, and 65 Gy respectively. In addition, no more than 2% of the PTV is to receive more than 84.7 Gy, and no less than 98% is to be covered by the prescribed dose (78 Gy). The femoral head doses are not mentioned in RTOG 0126. We applied the criteria for acceptable femoral head doses set out in Report 62 from the International Commission on Radiation Units and Measurements (ICRU),^(22)^ in which the volume that is covered by 52 Gy or more should be minimized (<5%).

### D. Dynamic arc techniques

In the present work, the DAT technique consisted of non‐shielded rectum bilateral arcs of ranges 36 – 136 degrees for left arcs and 226 – 326 degrees for right arcs to fit the PPO only, with a 3‐mm margin to MLC edges.^(2)^ The 2A‐DAT technique consisted of left and right 180‐degree dynamic arcs with a single isocenter. Each arc was designed to fit one of two separate symmetrical rhombi including the PPO.^(3)^ As a minor modification in the present work, we used a single isocenter instead of the two isocenters applied in the published 2A‐DAT so as to make patient setup easier.

In our previous work, the M‐DAT technique was introduced for the treatment of the prostate plus seminal vesicles with differential doses.^(5)^ However, in the present work, the technique is modified for treatment of the prostate only. Accordingly, in the M‐DAT technique used here, the two 350‐degree dynamic arcs are designed to fit the PPO only and to shield the VRV. However, there is no change in the design of the two wedged (enhanced dynamic 45‐degree angles of anteriorly oriented thick ends) conformal fields, which were symmetrical with respect to laterals (15 degrees downward), of low weighting, and assigned to cover the PPO only. The margins of the MLC aperture to the PPO for all fields were taken 5 mm in all directions except in the superior and inferior directions, where 8 mm was used. This arrangement was adequate to cover the PPO with 95% of the prescribed dose.

In the BW‐DAT technique, two bilateral pairs of 180‐degree dynamic arcs are generated automatically by the planning system to fit the PPO and to shield the VRV. To achieve dose distribution uniformity in the PPO, a hard 45‐degree wedge was applied with each bilateral pair of arcs in opposite orientations. This hard wedge was inserted at 0 gantry and collimator angles, such that its thin end at the left side for left arcs and at the right side for right arcs. The margins of the MLC aperture to the PPO for arcs in BW‐DAT were the same as those in M‐DAT. Table 1 shows the geometric arrangements and setup parameters of the dynamic arc techniques.

**Table 1:** Field arrangements and setup parameters of the dynamic arc techniques for the Varian 23 EX linear accelerator in the International Electrotechnical Commission scale

*Technique*	*Treatment fields*	*Arc ranges, field angles, and wedge orientations*	*Dynamic Volume conformed*	*MLC Volume shielded*
DAT	One left arc, one right arc	Right arc from 36 to 136 degrees, left arc from 226 to 326 degrees	PPO	None
2A‐DAT	One left arc, one right arc	Right arc from 0 to 180 degrees,	Two rhombi	None
BW‐DAT	Two opposed left wedged arcs, two opposed wedged right arcs	Right arcs from 0 to 180 degrees, wedged (45 degrees, thin end to the right); left arcs from 0 to 180 degrees, wedged (45 degrees, thin end to the left)	PPO	VRV
M‐DAT	Two opposed full arcs, two conformal wedged fields	Two arcs from 185 to 175 degrees (clockwise and counterclockwise), two lateral oblique fields (105 and 255 degrees) wedged (45 degrees, thick end to the anterior)	PPO	VRV

MLC=multileaf collimator; DAT=dynamic arc therapy; PPO=planning prostate‐only; 2A−DAT=two‐axis dynamic arc therapy; BW‐DAT=bilateral wedged dynamic arc; VRV=virtual rectum volume; M−DAT=modified dynamic arc therapy.

### E. Plan comparisons

For evaluation and comparison of all of the present techniques, the mean dose volume histograms (DVHs) of the PPO, RC, BL, FH, 3cmM, and Body–TV were calculated for all patients. All plans were normalized based on the DVHs to ensure that 95% of the PPO received 100% of the prescribed dose.

In the present publication, $V_{x\%}$ is the percentage of organ volume exceeding x% of the prescribed dose (78 Gy) and $D_{x\%}$ is the minimum dose to x% of the organ volume. Also, the minimum and maximum doses to any volume and the volumes covered by at least these doses are designated as ($D_{\text{min}}$ and $D_{\max}$) and (VDmin and VDmax) respectively.

For the comparison between the present and previously published IMRT data for the PPO, RC, and BL,^(20)^ we obtained the $D_{99\%}$, the dose inhomogeneity [DI, where $\text{DI}=(D_{\text{max}}-D_{99\%})\times 100/\;\text{prescribed dose}$] of the PPO, and in addition, the $D_{17\%}$, $D_{35\%}$, $V_{40\%}$, $V_{50\%}$, and $V_{60\%}$ of the RC, and the Dmax, $D_{25\%}$, and $V_{40\%}$ of the BL.

The $D_{\text{min}}$, VDmin, $D_{\text{max}}$, and VDmax of the PPO and the $D_{15\%}$, $D_{25\%}$, $D_{35\%}$, and $D_{50\%}$ both of the RC and of the BL were used to check and compare the tolerability of these doses and volumes to the RTOG 0126 criteria. The $D_{\max}$ and VDmax of the FH were used to inspect the acceptability of the FH protection according to ICRU Report 62 criteria.

The $D_{\text{max}}$, $V_{95\%}$, $V_{85\%}$, $V_{80\%}$, and $V_{70\%}$ of the 3cmM were used to compare the high‐dose protection to healthy tissue surrounding the PPO, but the $D_{\max}$ and $V_{10\%}$, $V_{20\%}$, and $V_{30\%}$ of the Body–TV were used to compare the dose protection to healthy tissue outside the TV.

The differences between any two techniques for doses to any percentage volume were calculated as [(D1−D2)/prescribed dose]×100, where *D*1 and *D*2 are the doses to the same volume in techniques 1 and 2 respectively.

### E. BW‐DAT plan verification

We verified M‐DAT in our previous publication.^(5)^ Applying the same procedure, we used the CT image sets of a cylindrical phantom (PTW T9193: PTW, Freiburg, Germany) and an Alderson Rando anthropomorphic phantom (PTW) to export and calculate in Eclipse BW‐DAT plans for all patients. A PinPoint ion chamber (PTW 31006, 0.015 cm^3^: PTW) located in the central axis of the cylindrical phantom was used to verify the calculated isocenter dose.

To verify the BW‐DAT plan, extended dose range film (EDR2: Eastman Kodak Company, Rochester, NY) was inserted axially into the Alderson phantom at the region of the prostate for each patient of interest. The dose distributions measured using film and calculated by Eclipse at the same levels were imported and compared using Varisoft software (ver. 3.1: PTW). The criterion used to evaluate the accuracy of the Eclipse calculations was the gamma index,^(23)^ with individual acceptance criteria of 3% dose difference (DD) and 3 mm distance to agreement (DTA). A quantitative analysis of the dose distribution comparison based on gamma reports was performed to show the percentage pixels in a scanned area 10×10 cm that exceeded the acceptance criteria (percentage failed pixels).

## III. RESULTS AND DISCUSSION

### A. Comparisons with previously published data

We first compared our results with previously published data to inspect the validity of the dose calculations from the 3D planning systems. Table 2 provides a quantitative comparison between data from the present study and published data for the IMRT technique.^(20)^ It shows that values of $D_{\text{max}}$, $D_{99\%}$, and DI for the PPO in the present work agree with those in published work with a maximum variation of 2.5%. The $D_{17\%}$, $D_{35\%}$, $V_{40\%}$, $V_{50\%}$, and $V_{60\%}$ of the RC in the two works are in agreement with a maximum variation of 8.2%. The $D_{\max}$ of the BL in the present IMRT data agrees with that previously published (variation: 1.2%). The $D_{25\%}$ and $V_{40\%}$ of the BL in the present work are lower by 18.7% and 8.1% respectively than those previously published. This difference could be the result of differences in the volumes of the BL and of proximity to the prostate. The dose to the FH was not mentioned in the previous publication.

**Table 2:** Quantitative comparison of present and published data^a^,^(20)^ for intensity‐modulated radiation therapy at doses of 78 Gy (present) and 74 Gy (published)

	*Planning prostate‐only target volume*			*Rectum*			*Bladder*	
	*Present*	*Published*		*Present*	*Published*		*Present*	*Published*
$D_{\text{max}}$ (%)	106.4±2.1	108.9±1.3	$D_{17\%}$ (%)	47.5±9.2	46.0±6.2	$D_{\text{max}}$ (%)	105.0±1.4	106.2±1.3
$D_{99\%}$ (%)	95.3±1.1	97.6±1.2	$D_{35\%}$ (%)	27.4±8.2	34.5±5.2	$D_{25\%}$ (%)	21.8±13.1	40.5±22.6
DI^b^(%)	11.1±1.9	11.3±1.7	$V_{40\%}$ (%)	20.5±5.6	12.4±4.1	$V_{40\%}$ (%)	12.2±10.2	20.3±10.2
			$V_{50\%}$ (%)	16.2±3.2	8.03±2.5			
			$V_{60\%}$ (%)	10.4±2.1	5.13±1.7			

a All values are mean±standard deviation.

b $(D_{\text{max}}-D_{99\%})\times 100/\text{prescribed dose}$.

$D_{\text{max}}=\text{maximum dose received by}\;1\;{\text{cm}}^{3}\text{in the volume of interest}$; $D_{x\%}=\;\text{minimum dose to}\;x\%\;\text{of the volume of interest}$; $V_{x\%}=\;\text{percentage of the volume of interest exceeding}\;x\%\;\text{of the prescribed dose}$.

Accordingly, our IMRT planning results agree with the previously published data.

A quantitative comparison between the present and previously published DAT and 2A‐DAT plans^(2)^ ^,^ ^(3)^ is not possible, because they are studied using different approaches. For example, the rectal and bladder walls are taken as organs at risk in the published DAT plan^(2)^; their doses therefore could not be compared with the present volumetric data for the BL and RC doses. The FH doses were not mentioned in those publications.^(2)^ ^,^ ^(3)^ Also, in the previously published 2A‐DAT plan,^(3)^ the prescribed dose to the prostate ranged from 60 Gy to 72 Gy (median: 70 Gy; mean: 67.9 Gy), and the RC doses were not tabulated, yielding inaccurate comparison results.

### B. Plan comparison

**Figure 1:**
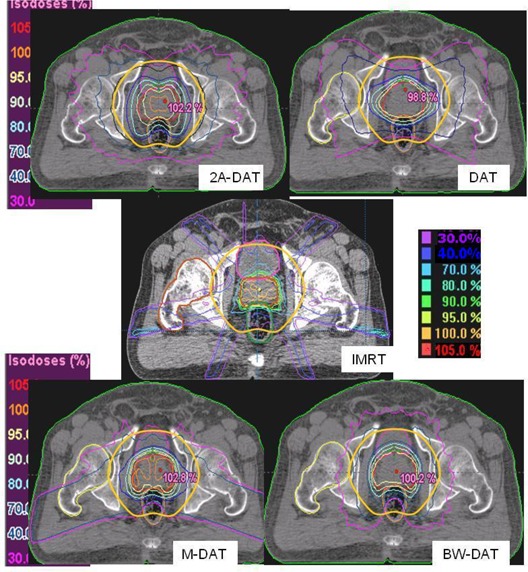
The isodose distributions of the five treatment techniques in a transverse section. The solid yellow contour is the 3‐cm margin around the planning prostate‐only target volume, which also defines the treatment volume. The blue contour inside rectum represents the virtual rectum volume (VRV). The white‐outlined shapes represented the two symmetrical rhombi, which are used in the construction of the dynamic multi‐leaf collimator with two‐axis dynamic arc therapy (2A‐DAT). DAT=dynamic arc therapy; BW‐DAT=bilateral wedged dynamic arc; M−DAT=modified dynamic arc therapy; IMRT=intensity‐modulated radiation therapy.

**Figure 2:**
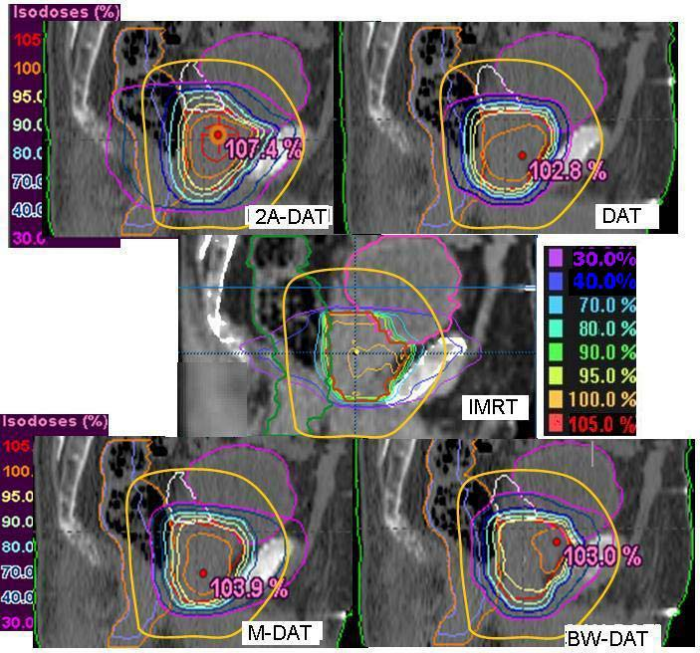
The isodose distributions of the five treatment techniques in the central sagittal section. The solid yellow contour is the 3‐cm margin around the planning prostate‐only target volume, which also defines the treatment volume. The blue contour inside rectum represents the virtual rectum volume (VRV). DAT=dynamic arc therapy; 2A−DAT=two‐axis dynamic arc therapy; BW‐DAT=bilateral wedged dynamic arc; M−DAT=modified dynamic arc therapy; IMRT=intensity‐modulated radiation therapy.

**Figure 3:**
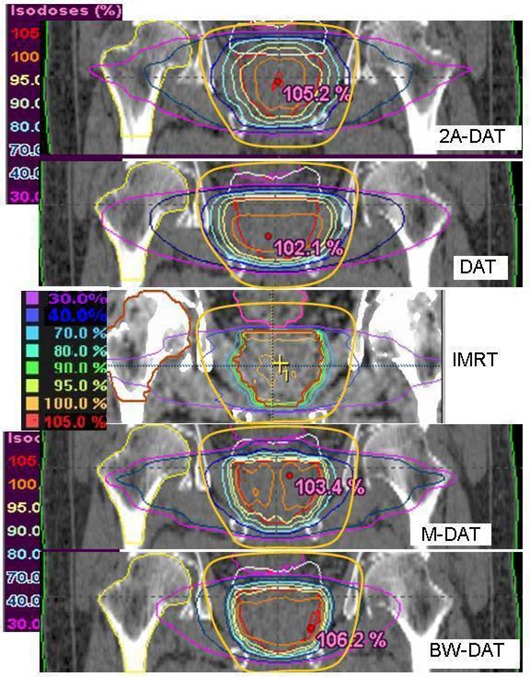
The isodose distributions of the five treatment techniques in the central coronal section. The solid yellow contour is the 3‐cm margin around the planning prostate‐only target volume, which also defines the treatment volume. The blue contour inside rectum represents the virtual rectum volume (VRV). DAT=dynamic arc therapy; 2A−DAT=two‐axis dynamic arc therapy; BW‐DAT=bilateral wedged dynamic arc; M−DAT=modified dynamic arc therapy; IMRT=intensity‐modulated radiation therapy.

Figs. 1, 2, and 3 show the isodose distributions that result from the IMRT, 2A‐DAT, DAT, M‐DAT, and BW‐DAT techniques in the transverse, sagittal, and coronal views. They show that IMRT and BW‐DAT are both better than the other techniques with respect to PPO coverage and 3cmM protection.

**Figure 4:**
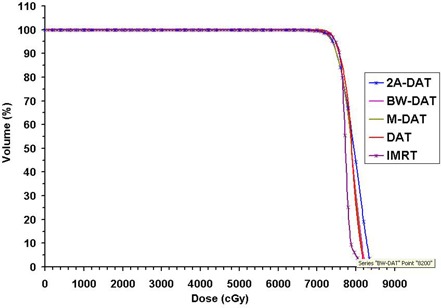
Plot, for the five treatment techniques, of the mean dose–volume histograms of the planning prostate‐only target volume for the 10 patients under study. DAT=dynamic arc therapy; 2A−DAT=two‐axis dynamic arc therapy; BW‐DAT=bilateral wedged dynamic arc; M−DAT=modified dynamic arc therapy; IMRT=intensity‐modulated radiation therapy.

Fig. 4 presents the mean DVHs for the PPO with the five techniques (2A‐DAT, BW‐DAT, M‐DAT, DAT and IMRT) for the 10 patients under study. This figure indicates that IMRT is better than the dynamic arc techniques with respect to PPO coverage. The quantitative comparisons of these DVHs, as shown in Table 3, indicates that the values of the $D_{\text{min}}$ and VDmin of the PPO are about 95% of the dose prescription and 100% respectively in all plans except for 2A‐DAT, where they are lower. Also, the $D_{\max}$ and VDmax to the PPO are acceptable in all plans according to RTOG 0126, except for 2A‐DAT, where they are higher. The results of 2A‐DAT therefore failed to respect the RTOG 0126 criteria under the present PPO dose normalization condition.

**Table 3:** Quantitative comparison of the mean dose–volume histograms of the planning prostate‐only with the five treatment techniques

	*DAT*	*2A‐DAT*	*M‐DAT*	*BW‐DAT*	*IMRT*
$D_{\text{min}}$ (Gy)	73.9±4.3	72.0±2.7	73.8±6.3	74.0±1.7	74.0±3.3
$D_{\text{max}}$ (Gy)	81.5±1.3	85.0±1.2	82.5±0.9	83.0±0.7	83.0±2.3
VDmin (%)	99.8±0.1	99.0±0.7	99.5±0.3	99.5±0.3	99.5±0.3
VDmax (%)	1.0±0.3	2.8±1.3	1.3±0.5	1.4±0.8	0.5±0.6

a All values are mean±standard deviation. The normalization condition was 100% of the planning prostate‐only covered by 95% of the prescribed dose.

DAT=dynamic arc therapy; 2A−DAT=two‐axis dynamic arc therapy; BW‐DAT=bilateral wedged dynamic arc; M−DAT=modified dynamic arc therapy; IMRT=intensity‐modulated radiation therapy; $D_{\text{min}}=\;\text{minimum dose to any volume}$; $D_{\text{max}}=\;\text{maximum dose to any volume}$; $V_{D\min}=\;\text{volume covered by the minimum dose}$; $V_{D\max}=\;\text{volume covered by the maximum dose}$.

**Figure 5:**
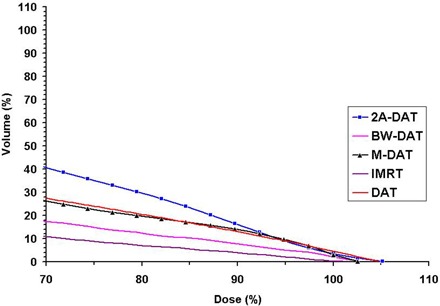
Plot, for the five treatment techniques, of the mean dose–volume histograms of the 3‐cm margin surrounding the planning prostate‐only for the 10 patients under study. DAT=dynamic arc therapy; 2A−DAT=two‐axis dynamic arc therapy; BW‐DAT=bilateral wedged dynamic arc; M−DAT=modified dynamic arc therapy; IMRT=intensity‐modulated radiation therapy.

With respect to dose protection to the 3cmM, the plot of its mean DVHs, as shown in Fig. 5, indicates that IMRT is the best, BW‐DAT is better than both M‐DAT and DAT, and 2A‐DAT is the worst. The superiority of dose conformity to the PPO in the case of IMRT is mainly attributable to the use of the micro‐MLC,^(20)^ but the inferiority of this conformity in 2A‐DAT is attributable to the use of the two rhomboid volumes instead of the PPO in the dynamic MLC shape design. Quantitatively, as Table 4 indicates, the $D_{\max}$ to the 3cmM in the case of IMRT is lower by 2.5% than that in both the BW‐DAT and M‐DAT techniques, but is lower by 5.0% than that in both the DAT and 2A‐DAT techniques.

**Table 4:** Quantitative comparison of the mean dose‐volume histograms of the 3‐cm margin (3cmM) surrounding the planning prostate‐only and the body minus the treatment volume (Body‐TV) with the five treatment techniques

	*DAT*	*2A‐DAT*	*3cmM M‐DAT*	*BW‐DAT*	*IMRT*		*DAT*	*2A‐DAT*	*Body‐TV M‐DAT*	*BW‐DAT*	*IMRT*
$D_{\max}(\%)$	105±2.6	105±2.7)	102.5±1.8	102.5±2.8	100±2.5	$D_{\max}(\%)$	60.5±9.4	56.5±8.7	52.5±7.2	42.5±2.8	87.5±12.0
*V* ^95%^ (%)	9.0±1.6	9.0±3.2	10.0±2.1	5.0±1.6	2.0±1.2	$V_{30\%}$ (%)	17.5±9.6	21.7±7.1	15.0±4.5	8.0±3.1	26.0±6.3
$V_{85\%}$ (%)	17.0±4.8	24.5±6.6	17.0±4.5	10.0±2.6	5.5±1.8	$V_{20\%}$ (%)	39.5±7.1	54.2±6.2	34.3±8.3	29.0±9.4	56.0±11.2
$V_{80\%}$ (%)	20.0±5.6	29.7±6.6	20.0±5.8	12.2±4.6	7.5±3.1	$V_{10\%}$ (%)	66.0±10.6	85.0±12.6	66.0±14.2	66.0±13.8	82.0±13.5
$V_{70\%}$ (%)	27.0±8.6	41.0±7.6	26.0±12.5	17.7±8.4	10.7±3.6						

a All values are mean±standard deviation.

DAT=dynamic arc therapy; 2A−DAT=two‐axis dynamic arc therapy; BW‐DAT=bilateral wedged dynamic arc; M−DAT=modified dynamic arc therapy; IMRT=intensity‐modulated radiation therapy; $D_{\text{max}}=\;\text{maximum dose to the volume of interest}$; $V_{x\%}=\;\text{percentage of the volume of interest exceeding}\;x\%\;\text{of the prescribed dose}(78\;\text{Gy})$.

The values of $V_{95\%}$, $V_{85\%}$, $V_{80\%}$, and $V_{70\%}$ in the case of IMRT are lower on average by 4.8% than those in BW‐DAT (with a maximum variation of 7.0%). Also, they are lower on average than those in M‐DAT, DAT, and 2A‐DAT by 11.8%, 11.8%, and 19.6% respectively, with maximum variations of 15.3%, 16.3%, and 30.3% respectively. In all cases, the maximum variations are noted with $V_{70\%}$, meaning that BW‐DAT is the technique closest to IMRT with respect to 3cmM protection.

**Figure 6:**
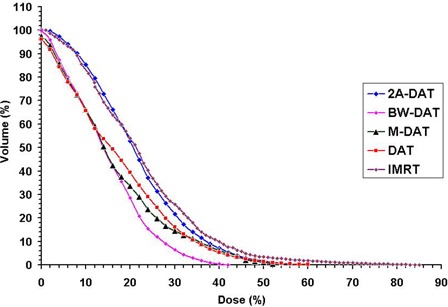
Plot, for the five treatment techniques, of the mean dose–volume histograms of the body minus the treatment volume for the 10 patients under study. DAT=dynamic arc therapy; 2A−DAT=two‐axis dynamic arc therapy; BW‐DAT=bilateral wedged dynamic arc; M−DAT=modified dynamic arc therapy; IMRT=intensity‐modulated radiation therapy.

On the other hand, we noted that significant regions of high dose (>70%) appear in the normal tissue outside of the treatment area (in the Body–TV) with IMRT, as shown in Fig. 1. Also as shown in Figs. 1, 2, and 3, we noted that the 40% isodose line covers a smaller area in the Body–TV with BW‐DAT than with the other techniques. The $D_{\max}$ to the Body–TV with BW‐DAT is 42.5%±2.8%, as shown in Table 4. This dose covered volumes of 8%±3.2% with IMRT, 4%±1.5% with DAT, and 6%±1.2% with both 2A‐DAT and M‐DAT, as determined from the quantitative analysis of the mean DVHs of the Body–TV illustrated in Fig. 6. Therefore, outside the treatment volume, significant volumes are covered by doses exceeding 33.15 Gy in all techniques except for BW‐DAT, where this volume is negligible. Also as shown in Table 4, the values of the $V_{20\%}$ and $V_{30\%}$ of the Body–TV in IMRT are higher than those in BW‐DAT, M‐DAT, 2A‐DAT, and DAT by 27% and 18%, 21.7% and 11.0%, 1.8% and 4.3%, and 16.5% and 8.5% respectively. The values of the $V_{10\%}$ in BW‐DAT, DAT, and M‐DAT are the same, but they are lower than those in IMRT and 2A‐DAT by 16.0% and 19.0% respectively. These results indicate that BW‐DAT is the superior technique with respect to Body–TV protection, which logically implies minimal radiotoxicity.

**Figure 7:**
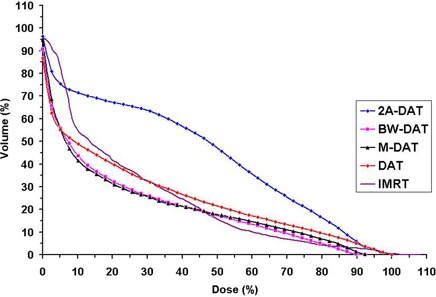
Plot, for the five treatment techniques, of the mean dose–volume histograms of the rectum for the 10 patients under study. DAT=dynamic arc therapy; 2A−DAT=two‐axis dynamic arc therapy; BW‐DAT=bilateral wedged dynamic arc; M−DAT=modified dynamic arc therapy; IMRT=intensity‐modulated radiation therapy.

Fig. 2, and also the mean DVHs of the RC presented in Fig. 7, indicate that the RC protection is comparable in the high‐dose region (>45Gy) in all techniques except for 2A‐DAT, but that BW‐DAT and M‐DAT are better than the other techniques in the lower dose regions. The RC protection in case of 2A‐DAT is the worst in the whole dose range.

Table 5 indicates that the $D_{15\%}$ of the RC in the case of IMRT is lower than that in BW‐DAT, M‐DAT, 2A‐DAT, and DAT by 7.3%, 10.3%, 33.0%, and 17.6% respectively. This finding is attributable to the superior dose conformity to the PPO in case of IMRT, which led to better high‐dose protection to the anterior rectal wall in that case. The $D_{25\%}$, $D_{35\%}$, and $D_{50\%}$ of the RC volume in the case of IMRT and DAT are comparable (maximum variation: 4.5%), as are those in M‐DAT and BW‐DAT (maximum variation: 1.5%). These doses in BW‐DAT are lower than those in IMRT by 8.7%, 10.6%, and 6.2% respectively, but they are quite lower than those in 2A‐DAT, given that the average variation is 41.6% (maximum: 44.0%). These superior low‐dose values to the RC in BW‐DAT and M‐DAT are attributable to complete MLC shielding of the

**Table 5:** Quantitative comparison of the mean dose‐volume histograms of the rectum and bladder with the five treatment techniques

	*DAT*	*2A‐DAT*	*Rectum M‐DAT*	*BW‐DAT*	*IMRT*	*DAT*	*2A‐DAT*	*Bladder M‐DAT*	*BW‐DAT*	*IMRT*
$D_{15\%}$ (Gy)	51.5±9.0	63.5±8.2	45.8±7.3	43.5±8.6	37.8±5.0	21.5±4.0	37.2±11.2	31.5±12.3	33.2±10.9	33.2±13.4
$D_{25\%}$ (Gy)	33.5±6.3	55.5±7.3	24.2±8.6	24.2±8.7	31.0±5.3	7.8±6.0	28.8±9.8	18.1±8.9	17.4±7.9	15.3±10.2
$D_{35\%}$ (Gy)	20.5±4.3	47.5±6.3	12.0±7.2	13.2±4.2	21.5±6.4	6.5±3.5	19.0±6.2	10.5±5.3	6.5±4.3	7.8±6.2
$D_{50\%}$ (Gy)	7.0±2.3	37.5±6.8	5.5±3.2	5.7±2.1	10.5±2.2	4.0±3.0	6.1±3.0	4.0±2.0	4.2±3.5	6.1±3.0

a All values are mean±standard deviation.

DAT=dynamic arc therapy; 2A−DAT=two‐axis dynamic arc therapy; BW‐DAT=bilateral wedged dynamic arc; M−DAT=modified dynamic arc therapy; IMRT=intensity‐modulated radiation therapy; $D_{x\%}=\text{minimum}$ dose to x% of the volume of interest.

posterior rectal wall, where VRV is delineated, in their dynamic arcs. These reductions in rectal doses may reduce chronic rectal injuries with these techniques, as suggested by several studies.^(^ ^24^ ^–^ ^26^ ^)^

**Figure 8:**
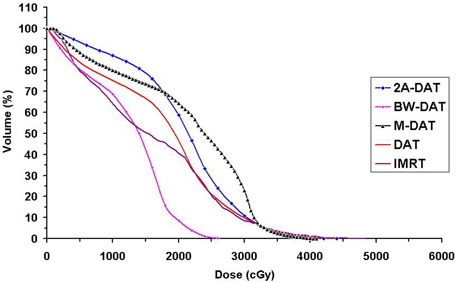
Plot, for the five treatment techniques, of the mean dose–volume histograms of the bladder (BL) for the 10 patients under study. DAT=dynamic arc therapy; 2A−DAT=two‐axis dynamic arc therapy; BW‐DAT=bilateral wedged dynamic arc; M−DAT=modified dynamic arc therapy; IMRT=intensity‐modulated radiation therapy.

As shown in Figs. 1 and 2, DAT is better than the other techniques with respect to BL dose protection. In comparison with the other techniques, DAT delivers minimal entrance anterior and exit posterior doses to the BL as a result of its relatively limited bilateral arc range, together with its favorable dose conformity to the PPO in the anterior direction. The mean DVHs of the BL presented in Fig. 8 indicate that the BL doses in IMRT, M‐DAT, and BW‐DAT are comparable, somewhat higher than those for DAT, and significantly lower than those for 2A‐DAT. The quantitative analysis of the doses to the BL as presented in Table 5 indicates that the $D_{15\%}$ and $D_{25\%}$ of the bladder in IMRT, M‐DAT, and BW‐DAT are comparable (maximum variation: 2.2% and 3.6% respectively), and that the mean values of each dose are lower than those in DAT by 14.3% and 11.7% respectively. However, the values of $D_{35\%}$ and $D_{50\%}$ in the four techniques are comparable, with maximum variations of 5.1% and 2.7% respectively. The $D_{15\%}$, $D_{25\%}$, $D_{35\%}$, and $D_{50\%}$ of the BL with DAT are lower than those with 2A‐DAT by 20.1%, 26.9%, 16.0%, and 2.7% respectively.

In all plans, as shown in Table 5, the results of the RC and BL doses respect the RTOG 0126 criteria. The $D_{15\%}$, $D_{25\%}$, $D_{35\%}$, and $D_{50\%}$ of the RC and BL with BW‐DAT are lower on average by 58.8% and 73.3% respectively than those recommended by RTOG 0126. Consequently, the BW‐DAT technique can be suggested for hypofractionated dose escalation regimes in the trials of 60 Gy in 20 fractions, 55 Gy in 20 fractions, or 52.5 Gy in 20 fractions for prostate cancer radiotherapy.^(^ ^27^ ^–^ ^29^ ^)^

**Figure 9:**
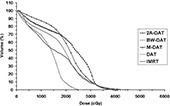
Plot, for the five treatment techniques, of the mean dose–volume histograms of the femoral head and neck for the 10 patients under study. DAT=dynamic arc therapy; 2A−DAT=two‐axis dynamic arc therapy; BW‐DAT=bilateral wedged dynamic arc; M−DAT=modified dynamic arc therapy; IMRT=intensity‐modulated radiation therapy.

Fig. 9 presents the mean DVHs of the FH for all plans. Because the $D_{\max}$ for the FHs for all patients are very close to each other (maximum variation: 3.8 Gy) with IMRT, only one is represented in Fig. 9. That figure shows that, as compared with the other techniques, BW‐DAT provided the most dose protection to the FH, because its $D_{\max}$ is 26.0±2.4 Gy, which is lower by 20.5 Gy on average than that in the other techniques. The results of the FH doses in all plans therefore respect the ICRU Report 62 criteria. Because the ranges of the bilateral arcs in BW‐DAT yield lower dose contributions to the FH than do those in DAT, the nonexistence of lateral beams in BW‐DAT (which is not the case in M‐DAT and IMRT) means that the dose gradient in lateral directions in BW‐DAT is lower than that in 2A‐DAT, as shown in Figs. 1 and 3.

Because the FH is completely included in the Body–TV and because the maximum dose to the Body–TV with BW‐DAT as calculated from Table 4 is 33.2±2.2 Gy, it is very difficult to exceed the recommended ICRU Report 62 dose tolerance (52 Gy) for the FH with the BW‐DAT technique. As a result, delineation of the FHs as organs at risk could be omitted.

The disadvantage of the 2A‐DAT technique is its lack of favorable dose conformity to the PPO, which yields small regions of lower dose inside the PPO and others of high dose outside it. As a result, the concave dose distributions did not satisfy their intended dose protection to the RC and BL. Nevertheless, our results for the 2A‐DAT technique respect the RTOG 0126 criteria for doses to the RC and BL, and the ICRU Report 62 criteria for the dose to the FHs, which make it an acceptable radiotherapy technique for the treatment of prostate cancer. However, it provides less protection to the BL and RC than do IMRT and the other dynamic arc techniques presented in this study.

### D. BW‐DAT plan verification

**Figure 10:**
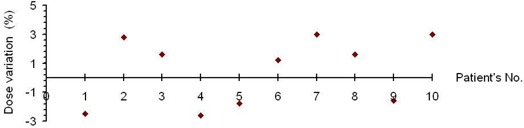
Percentage variation of the calculated and measured isocentric doses with bilateral wedged dynamic arc at the level of the prostate for the 10 patients under study.

The ion chamber measurements performed at the isocenter of the cylindrical phantom indicated that the measured doses agree with those calculated at the same point. Fig. 10 shows the percentage variation of the calculated and measured doses at the level of the prostate for all patients. The maximum deviation between the measured and calculated doses is 3.2%.

**Figure 11:**
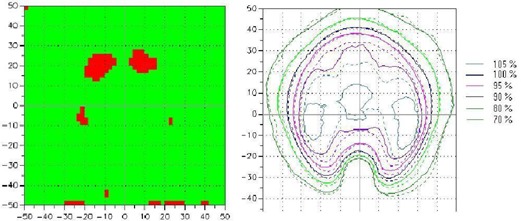
An example of the gamma distribution (left panel) and the measured and calculated dose distributions (right panel) with bilateral wedged dynamic arc for a scanned and calculated area of 10×10 cm (the scale of coordinate axes is 10 mm). The green areas indicate regions where pixels passed the gamma acceptance criteria (3% dose difference and 3 mm distance to agreement); red areas indicate regions where pixels failed. The continuous and dashed lines represent the measured and calculated dose distributions respectively.

**Figure 12:**
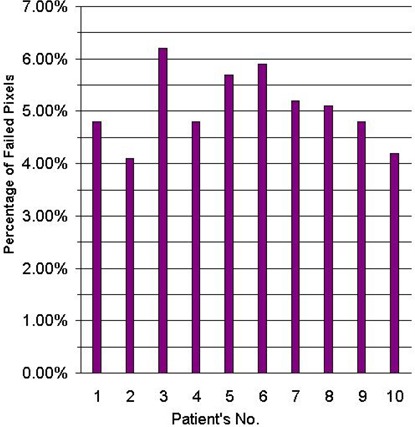
Percent failed pixels in a bilateral wedged dynamic arc dose distribution as compared with the gamma acceptance criteria (3% dose difference and 3 mm distance to agreement) for the prostate for the 10 patients under study.

Fig. 11 shows an example of gamma distributions and the measured and calculated dose distributions in the scanned area. Fig. 12 shows the percentage failed pixels in the scanned area for all patients. The percentage failed pixels does not exceed 6.5% with any patient. Consequently, it is easy to see that the isodose distribution calculated by Eclipse in the case of BW‐DAT is acceptable according to gamma index criteria (3% DD and 3 mm DTA).

## IV. CONCLUSIONS

The BW‐DAT, M‐DAT, and DAT techniques were found to provide the intended coverage of the prescribed dose to the prostate; however, the IMRT technique provides slightly better coverage. Compared with IMRT, BW‐DAT produces slightly less protection to the 3 cmM healthy tissue surrounding the prostate, but more than is seen with the other techniques. The dose gradient in the rest of the body in case of BW‐DAT is steeper than that seen with the other techniques. This finding permits the FHs to be ignored as organs at risk, because it is very difficult to exceed their dose tolerance with BW‐DAT.

Compared with IMRT and DAT, the BW‐DAT and M‐DAT techniques provide more dose protection to the posterior rectal wall. With respect to bladder protection, BW‐DAT and M‐DAT are comparable to IMRT, but DAT is the best. The 2A‐DAT technique performs worst in most of our comparisons, even though it satisfies the RTOG 0126 and ICRU Report 62 dose criteria for the organs at risk. Consequently, in addition to simplicity of dose calculation with the BW‐DAT technique, this technique provides the intended concave dose distribution for treatment of the prostate only. Compared with IMRT, it produces better dose protection to most of the rectum volume and the healthy tissue outside the treatment volume. As compared with the other forward‐planning dynamic arc techniques, it also produces the most favorable isodose distributions to the prostate and rectum.

## ACKNOWLEDGMENT

We thank our consultants and colleagues in the departments of Radiation Physics and Radiotherapy in Maadi Armed Forces Medical Compound.
